# AI-driven routing pipeline in software-defined networks using DQL: a mini review

**DOI:** 10.3389/frai.2025.1685155

**Published:** 2025-11-13

**Authors:** Deepthi Goteti, Vuyyuru Krishna Reddy

**Affiliations:** Department of Computer Science and Engineering, Koneru Lakshmaiah Education Foundation, Vijayawada, India

**Keywords:** software-defined networking, deep Q-learning, reinforcement learning, intelligent routing, fat-tree topology, quality of service

## Abstract

State-of-the-art data center networks are experiencing an increase in dynamic traffic. Even minor inefficiencies cause latency, congestion, and high costs. Software-defined networking (SDN) provides centralized programmability, but classical algorithms such as Dijkstra and Equal-Cost Multi-Path (ECMP) fall short because they cannot adapt in real time. To overcome this limitation, Reinforcement Learning (RL), particularly Q-learning, adds adaptability; however, scalability remains a challenge. DQL addresses this by using neural networks to approximate the Q-function, enabling SDN controllers to learn routing strategies directly from live network states. This Mini Review brings together recent DQL approaches for SDN. We examine architectures, algorithmic variants, and emulation environments (such as Mininet with Ryu). In addition, we introduce a structured taxonomy, with a practice-oriented synthesis of empirical trade-offs and deployment issues. The focus is on trade-offs, throughput, latency, and convergence. Reported studies show that DQL typically improves throughput by about 15–22 percent and reduces delays by roughly 10–12 percent compared with ECMP. These gains, however, come at the cost of longer training, inference delays, and scalability hurdles. Unlike prior surveys, this review makes three distinct contributions: a structured taxonomy, with a practice-oriented synthesis of empirical trade-offs and deployment issues. We also highlight emerging directions: federated learning, graph-based neural models, and explainable AI, which may help transition DQL from promising simulations to production-ready SDN solutions.

## 1 Introduction

Networks in data centers (DCNs) should be able to handle variable and unpredictable traffic loads, achieve low latency, high throughput, and efficient resource utilization.

Conventional hardware enclosed systems are frequently incapable of responding as dynamically as these demands would suggest. SDN has become a revolutionary concept because the control plane and the data plane have become disconnected, allowing control to be centralized, dynamically configured, and having a global view (Sezer et al., 2013).

This abstraction may enable the rapid deployment of new protocols, impose international policies, and allow networks to be optimally developed without requiring hardware changes. Most of the work in high-performance networks, including large-scale DCNs, is done by SDN controllers. They organize routing schemes, traffic management, and load balancing in order to meet high levels of performance. Continuing on this point, a sizable part of the SDN literature to date uses classical algorithms, such as the shortest path algorithm by Dijkstra (Yahya et al., 2015) or the Equal-Cost Multipath (ECMP) routing algorithm (Chiesa et al., 2014).

The relevant methodologies are effective in a static or predictable environment, but not very effective in a dynamic traffic environment (Yassen and Athab, 2025), which leads to congestion and underutilized links. ECMP allocates flows on equal-cost routes; however, it is unable to adjust to varying conditions dynamically. Goteti and Rasheed (2025) optimized their multipath routing algorithm in order to overcome this. It can flexibly cope with real-time traffic loads, topology, and performance metrics, which is more practice-intensive in the new version (Kamboj et al., 2023; Qin et al., 2024). Despite such advances, most studies remain limited to heuristic extensions or simulation-based proofs of concept, with little critical comparison of their real-world scalability, convergence costs, and impact on quality of service.

Existing surveys also tend to be descriptive, summarizing algorithms without analyzing their empirical trade-offs. This creates a gap that calls for a more analytical mini-review. To address these limitations, static and heuristic approaches have driven growing interest in Artificial Intelligence and Machine Learning (ML) for SDN routing (Xie et al., 2019; Nougnanke, 2021).

Reinforcement Learning (RL) is a data-driven approach that enables the optimization of policies through interaction with the environment (Sutton and Barto, 2018). Q-learning is a value-based RL algorithm that has been implemented in SDN routing (Jaafari et al., 2022; Piardi et al., 2019; Jayawardena et al., 2025), but its application is limited due to scaling problems in an ample state-action space. Building on these limitations, the next section outlines how Deep Q-Learning (DQL) operates within SDN, providing the conceptual background for the architectural analysis presented in later sections.

### 1.1 Contributions

In contrast to earlier surveys (which are largely summative), this Mini Review presents a systematic taxonomy of DQL-based SDN routing, summarizes empirical trade-offs across various studies, and provides a practice-oriented synthesis of deployment issues and implementation challenges. These orientations toward methodology and applicability make our work distinctive relative to previous reviews.

Taxonomy: a systematic taxonomy of DQL methods for SDN routing.

Empirical synthesis: Generalizes case-study results and measures throughput, delay, and convergence trade-offs.

Deployment issues: Critically assesses key systemic barriers, including scalability, real-time inference, security, and interpretability.

Practice-oriented roadmap: Describes emerging directions (federated, graph-based, explainable RL) needed to overcome the simulation-to-production gap in SDNs.

These contributions are new to DQL–SDN studies, as no previous Mini Review has integrated (i) a systematic taxonomy of methods, (ii) an empirical synthesis of case studies and benchmarks, and (iii) a deployment-focused critique.

The remainder of this paper is structured as follows: Section 2 provides background on AI, ML, and RL within SDN, including an overview of DQL; Section 3 describes the DQL frameworks and taxonomy; Section 4 discusses case studies and empirical findings; Section 5 outlines challenges, limitations, and future directions; and Section 6 presents the conclusion.

## 2 Artificial intelligence, machine learning, and deep Q-learning in software-defined networks

In actual SDN environments, traditional routing techniques frequently fail due to the complexity and layered structure of modern networks. Diverse network topologies, erratic traffic flows, and disparate quality-of-service priorities frequently cause fixed routing strategies to utilize network resources ineffectively. As a result, there has been a need for adaptive routing and policy optimization, which has led to the application of AI, particularly ML and RL, in SDN frameworks (Qin et al., 2024).

### 2.1 Foundations of reinforcement learning

Reinforcement Learning (RL), which typically operates as an independent branch of artificial intelligence, focuses on agents learning optimal actions by interacting with their environment (Sutton and Barto, 2018). RL works with an agent, environment, states, and rewards. In SDN, routing algorithms are considered intelligent agents. That selects the best paths for data to travel through the network. They examine the network’s layout and the amount of traffic currently flowing to inform their decisions. These agent’s primary goal is to work with strategies that maximize their performance, measured by factors such as the amount of data they can transmit simultaneously (throughput) or the speed at which the data reaches its destination (reduced delay) (Song et al., 2024).

Initially, Q-learning was the first method used to apply Reinforcement Learning (RL) in SDN. In Software-Defined Networking, routing algorithms determine the best paths for data by assessing the network’s layout and traffic conditions. The goal is to enhance performance by achieving higher throughput and lower delay (Sharma et al., 2024).

Recent work has started adapting these methods to real-world scenarios. Lin et al. (2025) introduced a deep learning method that considers performance factors such as bandwidth, which is in high demand in routing. This is achieved by predicting bandwidth needs, which helps prevent flow starvation during periods of heavy traffic. Likewise, the DQQS framework targets both performance and security in SDN–IoT environments. It detects possible attacks and assesses the quality of service. The goal of this research is to improve network performance by strengthening resistance to malicious traffic (Zabeehullah et al., 2024). These developments demonstrate how routing research is evolving from theory to workable, multi-objective solutions tailored to specific domains, taking these cases into account.

Recent advances in DRL for SDN Routing: Over the past 2 years, the study of DRL-based routing has progressed rapidly, particularly in the form of hybrid architectures that enhance the modeling of deep neural networks and policy optimization. In the research by He et al. (2024), it was proposed that a DRL scheme could be strengthened with the help of a graph neural network to help represent the topological dependencies in SDN topologies. Wang et al. (2024) proposed a dynamic-loaded DRL algorithm that uses a fuzzy-logic-based approach, ensuring latency and bandwidth constraints are met. Alenazi (2024) described a deep-Q-learning framework, which significantly improves both the QoS metrics (packet-delivery ratio and throughput). On the same note, Sensors (2022) and Bohrium (2024) presented ensemble- and transformer-based versions of DQL for scalable load balancing and routing. The subsequent advances indicate how the rule-based/tabular RL models gave way to architecture-sensitive, data-driven control in the present SDNs.

These recent innovations in DRL-driven routing mark a crucial transition point, linking earlier reinforcement-learning approaches with the broader integration of machine-learning techniques discussed in the following subsection.

### 2.2 Machine learning in the SDN context

Researchers thoroughly examined and assessed a variety of machine learning approaches for SDN applications prior to beginning work on Reinforcement Learning (RL). Important subjects covered in this investigation include traffic prediction, intrusion prevention, flow classification, and anomaly detection. An interesting survey by Xie et al. (2019) detailed the application of supervised, unsupervised, and reinforcement learning models in the fields of traffic engineering and network security.

By enabling autonomous decision-making at the network edge, machine learning has been shown in numerous studies to significantly improve network performance and lessen the burden on controllers. Studies by Nougnanke (2021) and Wang et al. (2022) emphasize this capability. One significant benefit is the wealth of high-quality datasets made available by SDN telemetry and flow-level monitoring.

Faezi and Shirmarz (2023) showed that supervised machine learning is currently at the forefront of anomaly detection and traffic classification. On the other hand, Reinforcement Learning and DQL are gaining traction in load balancing and adaptive routing.

Additionally, they noted that many assessments still make use of testbeds such as Mininet with Ryu or POX controllers, which indicates a promising direction for further research. Furthermore, machine learning successfully supports RL/DQL by providing predictive features that can enhance decision-making, such as anomaly detection and traffic load estimation.

Sha et al. (2023) claimed that a traffic prediction model based on machine learning aids in managing congestion in SDN using reinforcement learning techniques. Their work reflects the growing trend of integrating multiple AI methods in network control and demonstrates how combining adaptive decision-making with predictive analytics can improve routing efficiency.

### 2.3 Overview of deep Q-learning (DQL)

Consequently, DQL has emerged as a methodology applied to SDN to enable adaptive routing solutions by integrating deep neural networks with reinforcement learning. Neural architectures approximate the Q-function by training to take the optimal course of action based on the feedback received (Zhang et al., 2023; Sharma et al., 2024; Song et al., 2024; Liu et al., 2021; Fu et al., 2020).

DQL is an extension of classical Q-learning that incorporates deep neural networks to approximate the optimal action-value function Q(s, a), enabling agents to make decisions in large and continuous states. By selecting routing actions under the guidance of a learned policy, the objective is to maximize the long-term cumulative reward (Hasselt et al., 2016). A Q-network obtains a prediction of the expected cumulative reward of each possible routing choice, rather than storing the Q-values in a table. Two core mechanisms are experience replay and target network to stabilize learning and mitigate oscillations common in tabular RL. Within SDN, the DQL agent continuously observes network telemetry (e.g., link utilization, delay, queue length) and updates routing rules in real time through the controller to maximize throughput and minimize congestion. Empirical studies confirm that DQL improves throughput by 15–25 percent and reduces end-to-end delay compared with ECMP or Dijkstra-based routing (Fu et al., 2020; Liu et al., 2021; Sharma et al., 2024; Aguirre-Sánchez et al. 2024). These capabilities are improved in new hybrid models: He et al. (2024) use graph neural networks to make topology-aware decisions, Wang et al. (2024) use fuzzy-logic layers to address latency and bandwidth requirements, and Li et al. (2025) use graph-transformer architectures to achieve robust and scalable routing. These advances illustrate DQL’s maturity as an intelligent control framework, forming the conceptual foundation for the architectural analysis presented in subsequent sections.

This workflow utilizes the DQL agent, which evaluates routing activity based on telemetry information processed by machine-learning predictors that approximate traffic conditions. These observations help the agent refresh the SDN controller, which consequently enforces the learned flow rules in the forwarding plane.

**Figure 1:**
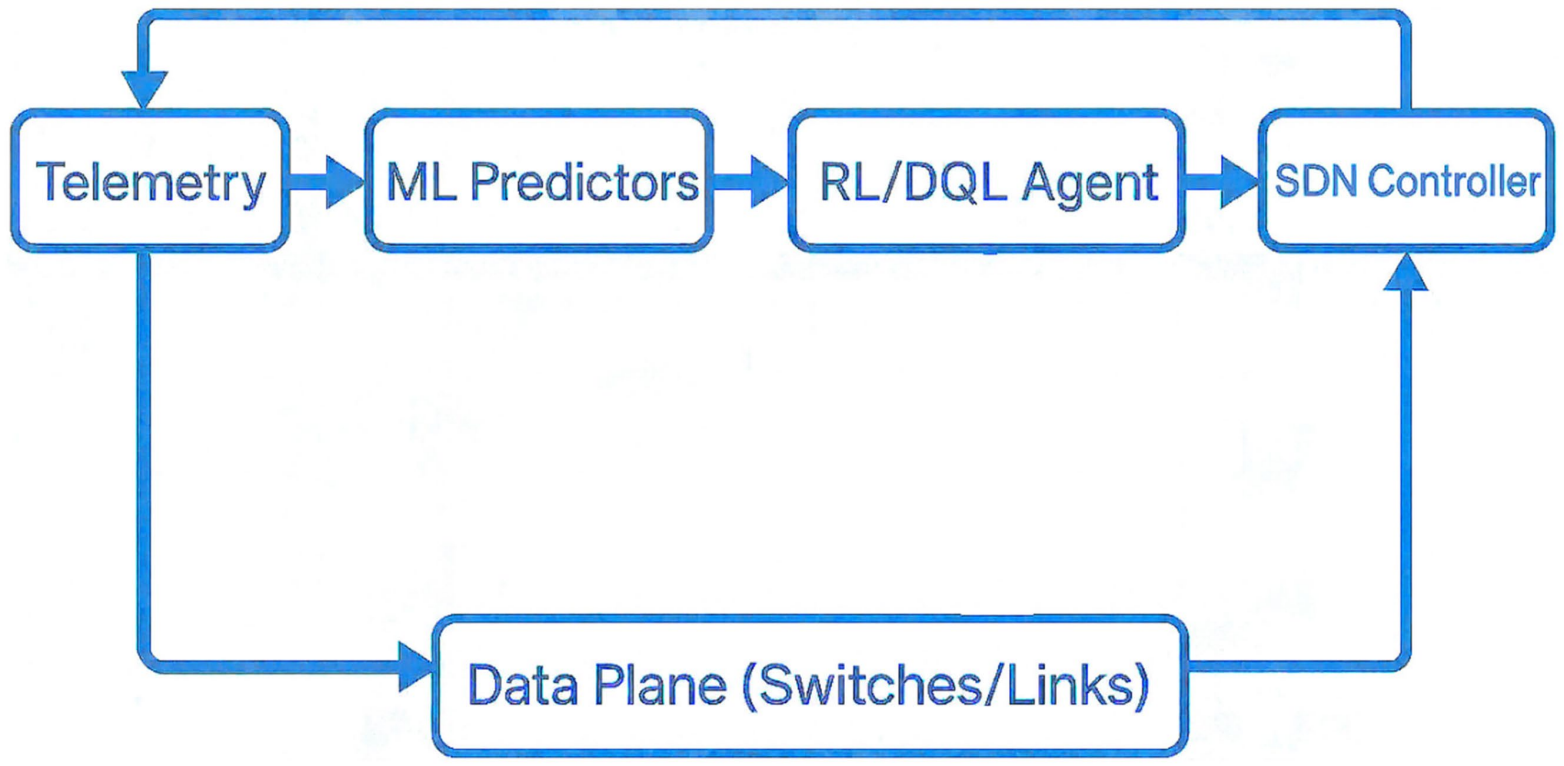
AI-driven SDN routing pipeline.

This setup allows finer-grained, real-time routing decisions in complex network topologies such as Fat-Tree data centers. Figure 1 illustrates this process: telemetry data are collected, analyzed by ML predictors, optimized through RL/DQL agents, and implemented by the SDN controller for adaptive routing. The signal originates at the Data Plane, flows through Telemetry/ML predictors to the DQL Agent, then to the SDN Controller, which enforces actions back in the Data Plane.

Practical studies demonstrate a clear trade-off. DQL consistently improves throughput and reduces delay; however, it also requires longer training and introduces inference latency. Measuring these trade-offs is essential—without it, progress from research prototypes to production-ready SDN systems remains difficult.

### 2.4 Transition to deep reinforcement learning (DRL)

**Figure 2:**
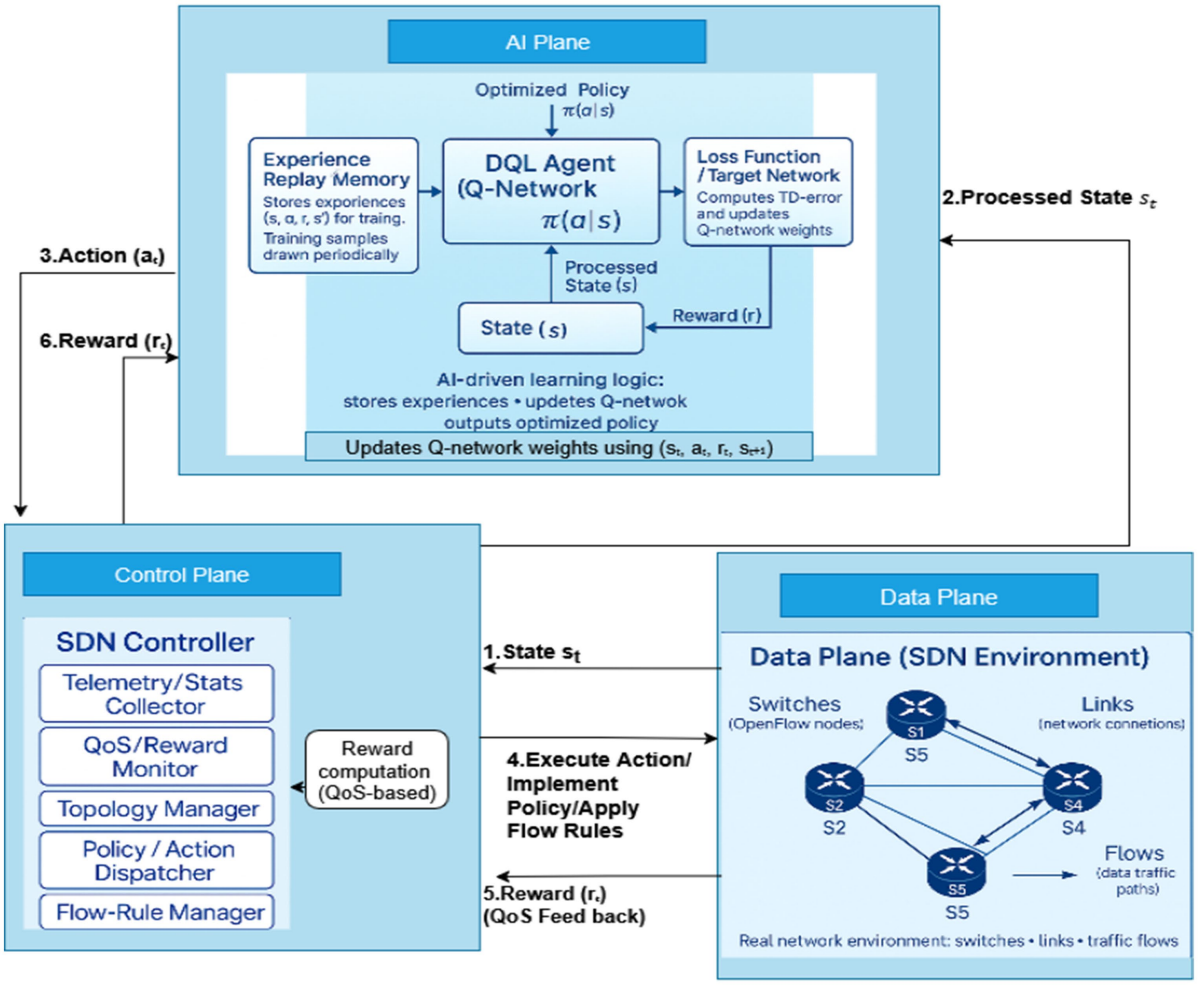
Architecture of a deep Q-learning-powered SDN framework.

DRL, which generalizes algorithms such as DQL, extends classical Reinforcement Learning to manage large and dynamic SDN environments. And it goes beyond the limits of classical Reinforcement Learning in managing large SDN environments. Instead of relying on static lookup tables, DRL uses deep neural networks to approximate the Q-function. Building on early breakthroughs in Deep Q-Networks (DQN), it has proven especially effective in handling high-dimensional and constantly changing environments (Silver, 2016; Arulkumaran et al., 2017; Rikhtegar et al., 2021). Within SDN, DRL enables the controller to adapt routing decisions in real time, considering factors such as bandwidth, latency, and packet loss. This makes the system context-aware and capable of self-optimizing control logic that responds quickly to topology changes or sudden traffic anomalies (Song et al., 2024). Building on these DRL foundations, Figure 2 illustrates how DQL integrates neural networks with classical Q-learning to enable adaptive, topology-aware routing within SDN environments.

In SDN, DRL allows the controller to modify routing choices. This enables the system to be context-aware and capable of self-optimizing control logic that can quickly adapt to topology changes or unexpected traffic anomalies (Song et al., 2024). The proposed DQL-based SDN framework (as shown in Figure 2) builds on this principle by integrating neural networks with classical Q-learning to emulate the action–value function and enable adaptive and efficient routing within large and dynamic network systems. Recent executions go beyond the traditional DQL and incorporate auxiliary learning modules. As an example, He et al. (2024) use fuzzy-logic inference to stabilize a learning process with variable loads, Wang et al. (2024) integrate graph neural networks with topology-aware perception, and Alenazi (2024) improves the reward design to enhance end-to-end QoS. In a similar manner, Bohrium (2024) uses transformer attention to facilitate the long-range flow correlation modeling. All these improvements indicate that DQL is becoming a multi-objective framework, which is modular and not based on a single algorithm model. The architecture consists of three collaborating planes—the Data Plane, Control Plane, and AI Decision Plane—which operate in a closed feedback loop to achieve continuous learning and optimization.

The process begins in the Data Plane (Forwarding Plane), where switches and links generate real-time telemetry and state information, including flow statistics, link utilization, and delay. The information is relayed to the SDN Controller in the Control Plane, which aggregates and preprocesses the data before forwarding it to the DQL Agent in the AI Decision Plane. The agent uses a policy network [π(a|s)] and an experience replay buffer to learn an optimal policy through the interaction tuple (s_t_, a_t_, r_t_, s_t+1_).

Once an optimal action is determined, the Control Plane translates the DQL output into network-level configurations, such as flow-rule updates or path adjustments, which are applied in the Data Plane. The resulting network performance measures (i.e., throughput, delay, and packet loss) are tracked by the QoS and Reward Module in the Control Plane and then converted into a reward signal (r_t_).

This reward signal leads to the updating of the Q-network, completing the closed-loop learning process. The interaction within the system enables continuous adaptation and self-optimization across heterogeneous SDN environments. Machine learning provides predictive insights, while reinforcement learning enables adaptive control. Their combination in DQL creates a real-time, self-evolving optimization framework that enhances routing intelligence, scalability, and responsiveness. Section 3 further elaborates on how this framework extends to deliver robust and scalable solutions for adaptive SDN routing.

### 2.5 Applications and research trends

The graph-based neural network with RL has been used in dynamic routing in smart grid-enabled SDNs to enhance fault tolerance and lower latency (Islam et al., 2023). Similarly, an adaptive routing system modeled on Q-learning realized significant improvements in the use of links and route discovery time using a multipath system (Goteti and Rasheed, 2025).

Tahi et al. (2024) employed machine learning classifiers with reinforcement learning to introduce an intelligent load-balanced method for SD-DCNs, which is utilized in path allocation and flow detection. Their comparative analysis with ECMP and heuristic schemes showed a better throughput and quality of service in Mininet and Ryu. This illustrates that the combination of ML and RL positively affects the load distribution in fat-tree data center networks (Yao et al., 2018).

Osman et al. (2023) designed a framework that links SDN controllers and traffic engineering deep learning models. One of the main characteristics of their work is the support for a multi-vendor environment, which is more practical for deployment. This brings DL-based SDN solutions closer to industry adoption. The design of such models should be very attentive. Mininet, as a tool that simulates network environments, as well as POX or Ryu to control network flow, are some of the standard tools used in this field. In the case of deep learning, many scientists are inclined to work with TensorFlow or PyTorch (Fontes et al., 2015; Wette et al., 2014; Abubakar et al., 2025). To stabilize the training of DQL, researchers have begun applying replay buffers and target networks; these techniques address convergence problems that frequently arise in previous studies of reinforcement learning (Hasselt et al., 2016).

Table 1 categorizes these approaches into several categories: temporal DQL, multi-agent DQL, graph-based models, quality-of-service- and security-aware DQL, federated learning, and hybrid solutions. Each option comes with trade-offs in adaptability, scalability, robustness, and feasibility. This overview illustrates the evolution of SDN routing research from classical Q-learning to advanced DQL methods, highlighting the directions that future intelligent networking may take. Taken together, these studies show a clear migration from heuristic routing to learning-based controllers capable of topology- and traffic-aware adaptation.

**Table 1:** Summary of AI techniques applied for routing optimization in SDN, highlighting the addressed problems, benefits, challenges, applications, and representative references.

AI techniques	Problem addressed	Benefits	Challenges	Applications	Key references
Q-Learning (Tabular)	Static routing; poor adaptability under traffic variation	Simple implementation; interpretable	Poor scalability; fails in high-dimensional state spaces	Basic SDN routing; testbeds	Piardi et al. (2019), Jaafari et al. (2022)
DQL	High-dimensional state-action routing in dynamic networks	Learns optimal paths; adapts to topology and traffic conditions	Slow convergence; requires extensive training and GPU resources	Fat-Tree, large-scale data center routing	Fu et al. (2020), Liu et al. (2021), Lin et al. (2025)
Temporal DQL	Lack of context in standard DQL; limited historical awareness	Uses traffic history; better long-term decision-making	State-space grows with memory; delayed convergence	Load balancing in hierarchical networks	Sharma et al. (2024)
Multi-Agent DQL	Single-agent models struggle in large or segmented networks	Parallel decision-making; scalable across multiple domains	Synchronization and stability of distributed learning	Distributed SDN controllers	Wang et al. (2019), Wang et al. (2022)
Graph-based DQL	Inability to model topology explicitly using flat features	Better generalization; scalable with changing topologies	Requires graph neural network integration; complex reward design	Topology-aware routing; Smart city networks	Islam et al. (2023); Li et al. (2025); He et al. (2024)
QoS-Aware DQL	Inability to prioritize latency-sensitive flows	Integrates QoS metrics into reward; better real-time traffic handling	Needs classification of flows; fairness trade-offs	VoIP, video conferencing over SDN	Song et al. (2024); Qin et al. (2024); Alenazi (2024)
Federated DQL	Privacy concerns and centralization limits	Decentralized training; local data utilization	Communication overhead; non-IID data between agents	Inter-domain SDN; federated learning in telecom	Suh et al. (2022)
Secure DQL (Adversarial Resilience)	Vulnerability to poisoning and reward manipulation attacks	Improved robustness; safer exploration policies	Detection overhead; attack model generalization	Industrial IoT, critical infrastructure SDNs	Zabeehullah et al. (2024); Nguyen et al. (2024)
Graph/Transformer Hybrid DQL	Lack of temporal + structural learning	Captures spatial and temporal patterns	Expensive training	Expensive training and high computational cost	Li et al. (2025); Bohrium (2024)
Edge/Fog-based DQL	High latency in centralized training	Lightweight inference at network edge	Limited resources; sync overhead	SDN-DCN; fog/edge IoT	Osman et al. (2023), Tahi et al. (2024)
Ensemble DQL	Instability in single DQL models under diverse traffic	Improves generalization and convergence stability through model ensembles	Requires ensemble coordination; increased compute cost	Optical transport networks; load balancing	Sensors (2022)

### 2.6 Challenges and considerations

Despite strong results, using ML and DRL in SDN faces several challenges:

- Training complexity: Mao et al. (2018) claim that deep learning models require significant computing power. Some models, such as lightweight edge agents and model compression, reduce this load (Ma et al., 2022).
- Data quality: Effective training requires fine-grained, real-time flow data. Inadequate telemetry lowers accuracy (Faezi and Shirmarz, 2023). Hybrid ML–RL methods fill in missing flow features, providing RL agents with better input.
- Controller overhead: DRL must respond quickly to avoid slowing the control plane (Kokila et al., 2025). Policy caching and distributing DRL across controllers lower latency and prevent bottlenecks (Wang et al., 2022).
- Scalability: Large topologies cause exponential state-space growth (Al-Fares et al., 2008). State abstraction, hierarchical DQL, and graph-based DRL improve scalability (Li et al., 2025).
- Deployment gap: Many designs remain at the simulation stage. Real-world use across multi-vendor SDN systems is still limited (Osman et al., 2023). Interoperable, controller-agnostic AI modules are needed.

Cutting-edge, recently developed methods such as distributed control systems, edge learning, and federated frameworks can solve many of the aforementioned problems. These methods promise improved scalability, performance, and privacy, even though the shortcomings of current systems underscore the need for more adaptive and scalable learning-based routing frameworks. Building on these recent DRL advances, the next section focuses on DQL as the core framework for intelligent, adaptive routing in SDN.

## 3 Deep Q-learning for intelligent routing in SDN

As outlined in the previous section, recent DRL-based routing frameworks have significantly expanded the scope of DQL, making it a practical and scalable solution for intelligent routing in Software-Defined Networks (SDNs). Conventional Reinforcement Learning methods, including Q-learning, are limited when applied to high-dimensional and dynamic network setups. DQL has been used to solve these issues by researchers. DQL combines deep neural networks and Reinforcement Learning to support more scalable and instant decision-making.

SDN has configurations and traffic patterns that are continuously evolving. In such vital activities as intelligent path selection, congestion control, and enhancing QoS, DQL is an efficient approach (Pham et al., 2021).

Unlike traditional Q-learning, which struggles with large sets of possible actions, DQL uses neural networks that learn from data. Because DQL can adapt to various network conditions, it is particularly beneficial for complex systems such as the Fat-Tree topology (Al-Fares et al., 2008).

### 3.1 DQL for routing optimization

DQL was tested in several SDN routing studies. Early research integrated DQL into an SDN controller, allowing it to adapt to varying link states. As a result, packet loss dropped, and end-to-end delay also decreased compared with static routing (Fu et al., 2020).

DRL-R is a more sophisticated variant to which the concept can be applied to the data centers, resulting in better load balancing, reduced flow completion time, and throughput in Fat-Tree networks (Liu et al., 2021; Tang et al., 2023). DQL variants that are dependent on the history of traffic conditions enhance the utilisation of the links and also reduce the congestion persistence in traffic situations during high loads (Sharma et al., 2024).

A large proportion of the implementations have been tested in emulation using Mininet, along with controllers such as Ryu or POX. DQL agents are written using either Python or TensorFlow and submit requests to the controller through APIs to calculate routing actions in real-time. The researchers use traffic generators such as Iperf and D-ITG to generate VoIP, video, or TCP / UDP traffic to mimic the actual workload.

These setups provide concrete validation of DQL’s performance improvements under realistic network stress conditions (Bhardwaj and Panda, 2022; Wirawan et al., 2024).

In order to resolve the contingent scalability problem, scholars have suggested hierarchical DQL agents applying state abstraction strategies as well as decentralized learning models that decentralize training to various controllers (Li et al., 2025; Suh et al., 2022). For real-time deployment, lightweight edge-based agents, model compression, and GPU acceleration have also been introduced to ensure that routing decisions meet millisecond-level latency requirements.

### 3.2 Adaptive routing in fat-tree topologies

Fat-Tree networks are widely used in large data centers because of their path diversity and redundancy (Al-Fares et al., 2008). However, conventional algorithms such as ECMP and Dijkstra often fail to exploit these advantages under dynamic traffic loads (McKeown et al., 2008). To address this gap, adaptive DQL models designed for Fat-Tree networks make use of traffic-aware features such as queue size, link delay, and flow priority to achieve better generalization performance (Li et al., 2022). Congestion-aware DQL agents have also been proposed to predict link congestion in advance, thereby reducing path switching and improving flow stability in emulated environments (Song et al., 2024; Nougnanke, 2021).

### 3.3 Multipath and QoS-aware extensions

DQL has been extended to multipath choice and QoS-concentrated routing goals to tackle the drawbacks of single-path routing. As one example, bandwidth- and jitter-conscious multipath DQL models can dynamically choose such paths that minimize shared bottlenecks, prioritizing latency-sensitive traffic especially (Zhang et al., 2023). Similarly, QoS-aware DQL models that incorporate service class prioritization into their reward function have achieved higher Quality of Experience (QoE) in heterogeneous traffic environments, which apply to 5G and multi-tenant data centers (Song et al., 2024; Qin et al., 2024). Distributed DQL agents have even been deployed in industrial SDN environments. In situational demands, where decentralized decision-making is demanded, reduce central controller overhead and increase fault resiliency (Wang et al., 2022).

### 3.4 Advanced architectures: graphs, transformers, and hybrid models

Recent works have investigated the integration of DQL with advanced neural designs to improve spatial and temporal reasoning (Wu et al., 2022). Graph-based DQL models utilize graph neural representations of the structural features of a network, while transformer-based DQL uses long sequences of state transitions to discover temporal dependencies. One such example is the Graph Transformer DQL model, which converges quickly and scales better when running SDN at scale (Li et al., 2025). Operationalizable tools such as Q-Optimizer build upon this advancement with a dual-agent coordination framework, modular architecture, and QoS-based feedback to uplift routing stability and responsiveness. With these advances, there are a number of limitations. The training run times remain lengthy, controller overhead grows with the size of the network, and model interpretability is poor, which still limits the use of DQL in practice in SDN production networks.

**Figure 3:**
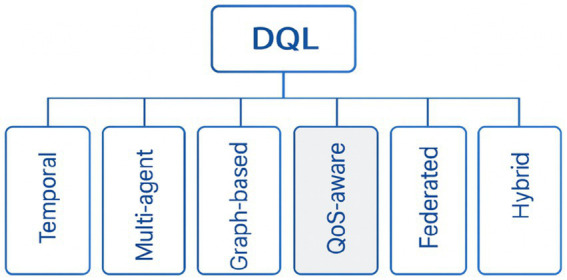
Taxonomy of deep Q-learning approaches for SDN routing.

These findings emphasize the importance of systematic evaluation, which is examined in Section 4 through the testing of DQL frameworks in Fat-Tree and other representative network topologies. To integrate the diversity of existing methods, we synthesized a conceptual taxonomy capturing the main design directions of DQL within SDN, as shown in Figure 3.

Recent literature aligns into six themes: temporal, multi-agent, graph-based, QoS-aware, federated, and hybrid architectures, all addressing one or more of the primary goals—scalability, adaptability, and service quality.

This unified perspective explains why various strategies converge to promote network intelligence and operational resilience. The following section builds on this synthesis by assessing how these DQL variants have been empirically validated across diverse experimental settings. These architectural refinements set up the performance analysis in Section 4, where we quantify DQL’s benefits and costs in representative topologies.

## 4 Performance evaluation in fat-tree topologies

DQL in SDN requires strong empirical validation. Results must extend beyond theory and demonstrate practical benefits. Fat-Tree topologies are widely used in data centers for this purpose because they provide multipath connectivity, redundancy, and scalability (Al-Fares et al., 2008). This section reviews the testing of DQL, focusing on topology selection, simulation tools, and performance metrics.

### 4.1 Fat-tree: a preferred topology

Fat-Tree networks are inherently redundant and feature multiple paths. They are of essential importance in data centers for load balancing and congestion-aware routing (McKeown et al., 2008). Also, they enable researchers to test routing strategies under erratic traffic conditions. The use of fat-tree networks is therefore employed as a standard to evaluate the network performance. They are used to assess fault recovery, quality of service, and routing among flow distribution. Owing to their flexible design, Fat-Tree topologies can be applied to compare adaptive methods, including DQL, to a baseline, including Dijkstra and ECMP (Wirawan et al., 2024; Li et al., 2022).

Empirical studies further confirm these advantages:

- These benefits are further proved by empirical research: In a k = 4 Fat-Tree, Fu et al. (2020) tested DQL and discovered roughly 30% reduce in delay and packet loss than ECMP.
- Liu et al. (2021) showed that DRL-R improved throughput by 20% and reduced flow completion time under heavy traffic.
- Zhang et al., (2022) demonstrated that congestion-sensitive DQL reduced the amount of packets drop and stabilized the flows under peak loads.
- DQL increased throughput by 15–22% percent and decreased delay by 10–12% percent over ECMP. These gains were associated with cost such as increased training time and increased inference overhead. The Fat-Tree has become the testbed of choice since it shows these trade-offs yet can be fairly compared to classical routing techniques.

### 4.2 Simulation tools and environment setup

Most evaluations of DQL in SDN utilize network emulation platforms combined with machine learning toolchains, which enable researchers to test under realistic workloads while maintaining control over experimental variables. Mininet is the most widely adopted emulator, particularly for deploying Fat-Tree topologies (k = 4, 6, 8) with native OpenFlow support. Fu et al. (2020) demonstrated that Mininet-based experiments achieved an average delay reduction of approximately 18% with DQL compared to ECMP in dynamic topologies.

On the control-plane side, the Ryu controller is often preferred because of its Python interface, which makes it straightforward to integrate with TensorFlow or PyTorch. According to Bhardwaj and Panda (2022), Ryu uses machine learning frameworks such as PyTorch and TensorFlow. Its usefulness in evaluating important metrics such as throughput, delay, and packet loss was validated by Wirawan et al. (2024). Combining Ryu and DQL improved throughput by almost 20% and decreased packet loss in mixed VoIP and video traffic, according to Zhang et al. (2023).

Traffic generators such as Iperf and D-ITG are widely used to emulate workloads, including bulk TCP transfers, VoIP calls, and HD video streams. Using these tools, Song et al. (2024) demonstrated that DQL preserved QoS by reducing jitter by up to 12% compared with static ECMP routing.

Fault tolerance has also been evaluated through failure-injection scenarios, such as link or switch crashes. Wang et al. (2022) found that distributed DQL reduced recovery time by about 25% and lowered controller overhead relative to centralized models.

Other controllers, including POX, ONOS, and OpenDaylight, have appeared in more limited studies. POX offers a lightweight option for proof-of-concept testing, whereas ONOS and OpenDaylight demonstrate the feasibility of industry-grade deployments.

Overall, these studies suggest that the performance of DQL is susceptible to the experimental setup, particularly the choice of controller, topology size, and traffic type. This suggests that, although the results are promising, they may not always generalize to all deployment environments.

### 4.3 Key metrics for evaluation

To judge how well DQLperforms in SDN routing, researchers often rely on QoS indicators. These highlight not only efficiency and reliability but also how effectively the system learns over time. These measures enable researchers to compare DQL with traditional approaches, such as ECMP and tabular Q-learning, and assess its performance under real operating conditions. In practical settings, Quality of Experience (QoE) indicators, such as latency, jitter, and packet loss, are often used to evaluate service quality, particularly in time-sensitive applications like VoIP and video streaming (Song et al., 2024).

Table 2 highlights the key indicators that demonstrate the relevance of the recent research. These investigations repeatedly demonstrate that DQL solutions are more reliable and flexible than standard solutions. Such findings represent a good step towards the more versatile and dependable SDN systems. Nevertheless, some limitations that remain should be noted. While improvements in throughput and reductions in delay are consistent, the training overhead can be 10 to 30 times greater than that observed with ECMP or heuristic approaches. Performance can vary widely depending on the controller used, such as Ryu or ONOS, and the scale of the network topology, including whether it is a k = 4 or k = 8 Fat-Tree. These trade-offs emphasize the importance of conducting systematic benchmarking across different environments.

**Table 2:** Performance metrics for evaluating DQL in SDN.

Metric	Description	Significance	Example findings/references
Throughput	Total data successfully delivered over time	Reflects link utilization and efficiency	DQL improved throughput by 18–22% vs. ECMP (Fu et al., 2020)
Packet Loss Rate	% of packets dropped due to congestion or link failure	Indicates network reliability	Loss reduced by ~15% under mixed workloads (Zhang et al., 2023)
Avg. End-to-End Delay	Time for a packet to travel from source to destination	Critical for real-time and QoS-sensitive apps	Temporal DQL cut delay by ~12% (Sharma et al., 2024)
Convergence Time	Time required for DQL to learn a stable routing policy	Demonstrates learning efficiency	DRL-R converged 30% faster than Q-learning (Liu et al., 2021)

From proof-of-concept implementations to large-scale, multi-controller systems, a comparative analysis of previous research shows a distinct trajectory in the development of DQL research within SDN. The baseline benefits of DQL in increasing throughput and decreasing delay under steady traffic conditions were validated by early frameworks (Fu et al., 2020; Liu et al., 2021). Federated and multi-agent models concentrated on distributed environments were detailed by Wang et al. (2022) and Suh et al. (2022), and examples of them were described by Sharma et al. (2024) and Li et al. (2025), and have temporal and hierarchical characteristics with increased convergence stability and faster policy adjustment. The failure to systematically implement convergence control weakens the system in terms of responsiveness and effectiveness. The developed trade-offs are evident: hierarchical DQL can be trained in an unstable manner, which can be improved with distributed and federated frameworks; however, this approach results in lower timeliness and responsiveness. Graph- or transformer-enhanced DQL delivers outstanding generalization across varying traffic conditions, but the processing requirements are enormous.

Single-agent DQL loses control precision and scales poorly, while control overhead remains high. Convergence toward system-level intelligence in DQL remains the missing element that unifies the findings described individually. This synthesis is critical for forming a comprehensive understanding of DQL, which, although currently advanced, remains iterative and limited. The impact of these findings helps shape the challenges addressed in the next section.

These persistent trades-offs between performance gains and training or inference overhead form the foundation of the challenges and open directions discussed in the following section.

## 5 Challenges, limitations, and future directions

Despite proven performance improvements, four systemic bottlenecks, scalability, efficiency, real-time inference, and trustworthiness (including security and interpretability), restrict the use of DQL in SDN. These issues are interrelated: lack of interpretability slows deployment in mission-critical networks, defenses against adversarial attacks add overhead, and larger models exacerbate inference delay. These restrictions and new solutions are explained in the ensuing subsections.

This section focuses on assessing DQL limitations in a novel, structured, and practice-oriented manner by combining empirical evidence from case studies, in contrast to previous surveys that only briefly discuss the challenges.

### 5.1 Scalability of learning models

Scalability is one of the biggest challenges when deploying DQL in large-scale SDN environments. As networks grow, the state and action spaces can expand dramatically. This requires deeper and wider neural networks to approximate value functions accurately. Fat-Tree and similar hierarchical topologies amplify this challenge because the number of possible routing combinations increases combinatorially (Chiesa et al., 2014).

At the moment, graph-based DRL frameworks naturally represent topological structure. Hierarchical DQL agents break down routing tasks into smaller, distinct subproblems, and state abstraction reduces the dimensionality of inputs (Zhang et al., 2023). Earlier surveys, particularly Prakoso et al. (2024), provided valuable taxonomies of reinforcement-learning-based routing but offered limited empirical comparison among scalability techniques. Recent work, including the Survey on Graph Neural Networks (Gkarmpounis et al., 2024), consolidates evidence that graph-based deep reinforcement learning provides the most scalable path when network topology varies dynamically, underscoring its structural adaptability and efficiency compared to flat feature-based models.

In contrast, this Mini Review integrates architectural reasoning with performance data to draw a comparative synthesis across three major scalability paradigms: hierarchical, graph-based, and federated/distributed frameworks.

Hierarchical DQL schemes converge more rapidly by breaking down learning into layer-wise subpolicies; researchers have found up to 20 to 25 percent faster convergence and reduced oscillation in larger networks in hierarchical DQL. DRA models based on graphs explicitly represent the network connectivity, which is more generalized and resilient to variable traffic conditions (Islam et al., 2023) but at a more expensive computational cost. By training local controllers concurrently (federated and distributed DQL) designs (Wang et al., 2022; Suh et al., 2022) can be more scalable and offer superior data privacy, but they add synchronization delays to the global system and cause uneven global convergence in cases of varying capabilities of individual controllers.

Collectively, the results outline a distinct trade-off space: hierarchical designs focus on convergence efficiency, graph-based designs on structural adaptability, and federated designs on horizontal scalability and privacy—though at the cost of communication overhead. Framing scalability in this comparative, quantitative way transforms it from a descriptive obstacle into a design space where efficiency, resource cost, and coordination latency can be balanced. This synthesis deepens understanding of when each paradigm is most effective and establishes the analytical basis for developing truly elastic, production-ready DQL systems for future SDN deployments.

Hybrid frameworks that combine hierarchical coordination and federated synchronization (e.g., Qin et al., 2024) may help balance this trade-offs by enhancing scalability while preserving rapid convergence. These comparative insights also frame the efficiency-focused and real-time inference discussions presented in the next sections.

### 5.2 Learning efficiency and convergence time

Thousands of episodes are normally required to achieve convergence. Methods such as Double DQN and experience replay enhance the stability and efficiency of policies (Sutton and Barto, 2018; Silver, 2016). Conversely, deterministic routing algorithms obtain immediate solutions, making DQL unpopular in networks where latency is a primary concern. Per Sharma et al. (2024), Priority Experience Replay reduced training episodes by almost 30 percent under heavy loads. Nevertheless, Piardi et al. (2019) demonstrated that cold-start delays could be minimized through transfer learning by reusing knowledge from related topologies. Moreover, techniques such as continuous learning and meta-learning are also evolving, allowing models to adapt to network variations over time without the need to restart the training process (Goteti and Rasheed, 2023).

DQL typically requires 1,000–5,000 training episodes to converge, whereas, according to empirical research, ECMP and Dijkstra converge almost immediately. This difference in efficiency continues to hinder adoption. Such inefficiency remains a significant obstacle to practical deployment.

### 5.3 Real-time inference constraints

The decisions made during routing in SDNs have to be performed in milliseconds, which large neural models, especially attention or transformer layers, cannot achieve. Bhardwaj and Panda (2022) identified that a combination of DQL and Ryu controller added 25% of latency to decision-making compared to the default ECMP. Wirawan et al. (2024) demonstrated that the lightweight pruning methods could achieve close to real-time performance. In real-world application strategies, such as edge-based agents, the use of a graphics card, and model compression, achieving accuracy, speed, and efficient resource consumption remains challenging. Practically, unoptimized DQL inference can add tens of milliseconds to decision latency, which is prohibitive for ultra-low-latency services, in contrast to ECMP, which has delays of less than 5 ms, which is prohibitive for ultra-low-latency services such as VoIP and 5G slices.

### 5.4 Security and robustness issues

During training and inference, DQL models are susceptible to poisoning and hostile manipulation. Attackers can inject malicious traffic to skew rewards or undermine regulations. Distributed DQL agents were especially susceptible to synchronized adversarial attacks, as Wang et al. (2022) pointed out (Islam et al., 2023; Wassie et al., 2024). Suggested defenses include anomaly detection, adversarial training, and reward defenses. Lightweight, attack-resistant models are therefore required, since these defenses increase computational costs and often reduce routing efficiency.

There is a way to secure DQL agents in real-world SDN deployments, thanks to recent developments such as the framework proposed by Nguyen et al. (2024), which demonstrates how adversarial attacks and defenses can be integrated into reinforcement learning to enhance policy robustness.

### 5.5 Integration and interoperability

The majority of DQL studies are limited in their applicability to heterogeneous, multi-controller deployments because of their reliance on Mininet simulations and single-vendor configurations. Phaneendra et al. (2024) highlighted the deficiency of controller-agnostic AI modules, while Deneke et al. (2024) demonstrated interoperability issues when scaling across multi-vendor SDN environments. Lightweight, standards-compliant models that support P4-based architectures, OpenFlow 1.3+, and hybrid cloud–edge deployments will be essential for future advancement.

### 5.6 Interpretability and transparency

Adoption and trust are hindered by the “black-box” nature of deep learning. Particularly in mission-critical industries such as healthcare and finance, operators must understand the rationale behind selecting specific routes. There have been proposals for explainable RL (XRL) techniques, such as saliency visualization and attention heatmaps (Xie et al., 2019; Li et al., 2025). By linking routing decisions to network states, these tools can increase compliance and trust. It is still unclear how to incorporate interpretability without compromising model accuracy.

To increase the openness of DQL routing rules, for example, causal explanation techniques such as the Causal State Distillation discussed by Lu et al. (2023) and the causal world models proposed by Yu et al. (2023) provide methods to connect agent choices to the underlying network states. A recent survey by Milani et al. (2025) provides a comprehensive categorization of explainable reinforcement learning methods, emphasizing their applicability to networking and other critical domains. Similarly, the XRL Survey (2025) classifies such techniques into agent-, reward-, state-, and task-based explanations, underscoring their relevance for trust-building in SDN environments.

### 5.7 Future scope: hybrid and federated architectures

In order to optimize scalability, responsiveness, energy efficiency, and interpretability, the next generation of DQLframeworks for SDN must move from isolated algorithmic experimentation to integrated, system-level design. Future research is now shifting from what algorithms can achieve to how those algorithms can coexist and cooperate within realistic, multi-controller environments. Building on recent progress, three prioritized and mutually reinforcing research pathways are emerging as the foundation for deployable, production-grade DQL-enabled networks.

- The highest priority is Federated-Hybrid Learning for Scalability and Privacy.

Scalability is unavoidably constrained by centralized training, which also presents data-sharing risks. Suh et al. (2022) showed how federated DRL achieves horizontal scalability with built-in privacy by enabling local controllers to train on private data while synchronizing only model parameters. Qin et al. (2024) advanced this principle for 5G network slicing, using multi-controller federated agents to balance load and enhance fault tolerance.

Future hybrid frameworks that fuse hierarchical coordination (for rapid local adaptation) with federated aggregation (for global policy consistency) can eliminate single-controller bottlenecks and make SDN learning architectures elastic and privacy-aware at scale.

- Lightweight and Energy-Efficient Inference for Real-Time Operation.

Even with federated training, high-complexity neural networks still hinder real-time decision-making. Emerging solutions, model pruning, quantization, distillation, adaptive compression, and edge/GPU/TPU acceleration, can cut inference latency from tens of milliseconds to near-instant responses while reducing power consumption. Integrating these optimizations with controller scheduling mechanisms will be essential for latency-sensitive environments such as industrial IoT, autonomous systems, and cloud-edge orchestration.

- Explainability, Security, and Trustworthiness in DQL Decision Making.

As DQL gains control authority over network routing, human interpretability and system reliability become critical. Explainable-RL techniques, including causal state visualization, attention-based policy mapping, and counterfactual analysis, can expose how routing decisions are derived and why specific actions are chosen. In parallel, adversarially robust and privacy-preserving training strategies (Nguyen et al., 2024) will guard against malicious manipulation of learning policies and ensure secure coordination among distributed agents.

#### 5.7.1 Integrated road map and outlook

Collectively, these three research directions outline a unified roadmap for the field:

- Federated-hybrid learning enables intelligence that is both scalable and privacy-aware.
- Energy efficiency and real-time responsiveness are achieved through lightweight inference.
- Transparency and operator trust are strengthened through explainable and secure DQL.

Together, they form the technical triad required to transform DQL from a promising research paradigm into a standards-compliant, deployable, and self-optimizing SDN control layer. To balance computational efficiency with learning depth and to create networks that are not only intelligent but also accountable and sustainable, these priorities should be integrated into cohesive frameworks in subsequent research.

This review therefore presents a strategic, practice-oriented research agenda for achieving intelligent, large-scale, and reliable SDN infrastructures by defining future work through these interconnected and prioritized pathways.

## 6 Discussion and conclusion

Integrating DQL into SDN enables enhanced network management, with real-time, adaptive routing that optimizes throughput, latency, and flow stability. Although the benefits are obvious, issues such as inference delay, scalability, and security have continued to stand as major barriers to deployment. This review highlights both the potential and practical limitations of DQL, underscoring the necessity for high-quality, reliable, and interoperable solutions to facilitate large-scale adoption.

Compared with earlier descriptive surveys, e.g., Prakoso et al. (2024), this Mini Review offers a practice-oriented and analytical point of view. In particular, it: (i) provides the first systematic taxonomy of DQL techniques to SDN; (ii) synthesizes the results of the recent research quantitatively to discover empirical trade-offs; and, (iii) critically analyzes the issues associated with deployment in the context of scalability, interoperability, and trust. The combination of these elements brings the review beyond summary in order to provide an evidence based roadmap to further development of DQL research into production ready and intelligent SDN systems. The next wave of research needs to be in the area of standardized benchmarking, cross-environment validation and integration of controllers to expedite the creation of self-organizing, resilient, and quality of service oriented network control systems. Overall, this work presents a structured taxonomy of DQL methods for SDN, providing a practice-oriented synthesis of empirical trade-offs and deployment challenges to guide future research and implementation.

## 7 Additional requirements

This Mini Review synthesizes prior research on Q-Learning, reinforcement learning, and DQLfor SDN routing and QoS optimization, in line with the Frontiers in Artificial Intelligence guidelines for Mini Reviews. It provides a focused overview, includes only peer-reviewed and publicly available data, and maintains a balanced, unbiased tone. The review outlines future directions like graph-based models, federated training, hybrid agents, and explainable RL. While critically examining methodologies, empirical findings, and open challenges (such as scalability, convergence, and real-time deployment). Researchers studying AI/ML and networking professionals working on 5G/6G networks and next-generation data centers will find the manuscript useful.
